# Anti-Inflammatory and Antioxidant Effects of Anthocyanins of *Trifolium pratense* (Red Clover) in Lipopolysaccharide-Stimulated RAW-267.4 Macrophages

**DOI:** 10.3390/nu12041089

**Published:** 2020-04-15

**Authors:** Sang Gil Lee, Cindi R. Brownmiller, Sun-Ok Lee, Hye Won Kang

**Affiliations:** 1 Department of Food Science and Nutrition, Pukyong National University, Busan 48513, Korea; Sglee1125@pknu.ac.kr; 2 Department of Food Science, University of Arkansas, Fayetteville, AR 72704, USA; cbrownm@uark.edu (C.R.B.); sunok@uark.edu (S.-O.L.); 3 Food and Nutritional Sciences, Department of Family and Consumer Sciences, North Carolina Agricultural and Technical State University, Greensboro, NC 27411, USA

**Keywords:** red clover, anthocyanins, anti-inflammation, antioxidation

## Abstract

Red clover (*Trifolium pratense*) possesses various dietary compounds that improve human health. However, the functions of anthocyanins in red clover remain unclear. Here we examined anti-inflammatory and antioxidant effects of red clover extract (RC) and red clover anthocyanins fraction (RCA) using lipopolysaccharide (LPS)-treated RAW 264.7 macrophages and identified dietary compounds. RC and RCA suppressed LPS-induced expression of genes such as tumor necrosis factor (*TNF*)*α*, interleukin (*IL*)*1β*, inducible nitric oxide synthase (*iNOS*), monocyte chemoattractant protein (*MCP*)1, and cyclooxygenase (*COX*)*2*. LPS-stimulated intracellular reactive oxygen species (ROS) production also was prevented by both RC and RCA. NADPH oxidase 1 (NOX1) gene and phosphorylation of p47^phox^ of NOX1 that were increased by LPS were inhibited in the cells treated with RCA. LPS-stimulated nuclear factor erythroid 2-related factor 2 (*NRF2*) gene expression and nuclear translocation of nuclear factor kappa B (NF-kB) subunit p65 were suppressed together with reduced iNOS and COX2 proteins by RCA. Additionally, 27 polyphenols and 7 anthocyanins from RC were identified and quantified. In conclusion, RC, especially RCA, exerted anti-inflammatory and anti-oxidative activities in vitro by regulating NF-κB and NRF2 signaling pathways, suggesting that anthocyanins in red clover are the potential candidates to reduce inflammation and oxidative stress.

## 1. Introduction

*Trifolium pratense* (red clover), which belongs to the bean family Fabaceae (or Leguminosae), is a medicinal plant that improves various health conditions such as asthma, whooping cough, cancer, and gout [1]. Phytoestrogenic isoflavones of red clover such as daidzein, genistein, biochain A, and formononetin improved menopausal symptoms and vaginal cytology on menopausal women [2,3,4,5]. Although red clover is a rich source of isoflavones, it is expected to have anthocyanins due to its color. However, anthocyanins of red clover are not identified. Anthocyanins are the plants’ pigments ranging from orange and red to purple and blue, which is classified as a subgroup of flavonoids with various health benefits such as antioxidants and anti-inflammation [6,7,8]. Inflammation is a response against infection, illness, and injury by producing cytokines such as tumor necrosis factor (TNF)α, interleukin (IL)1β, and IL6, and further by generating reactive oxygen species (ROS) [9]. Moreover, inflammation and oxidative stress are positively correlated with the development of chronic diseases such as obesity, diabetes, and cardiovascular disease, which attracts scientists to find new sources that have anti-inflammatory and antioxidant effects. Of natural sources, anthocyanins have been suggested as the potential candidate to ameliorate health issues related to inflammation and oxidative stress [10]. Nuclear factor kappa B (NF-κB) is a major regulator for anti-inflammatory and antioxidant effects of anthocyanins [11]. Malvidin-3-glucoside, a major anthocyanin of blueberries and grapes, suppressed TNFα- and IL4-stimulated inflammatory markers in human umbilical vein endothelial cells and peripheral blood mononuclear cells by inhibiting nuclear translocation of p65 of NF-κB [12,13]. Cyanidin-3-glucoside reduced production of nitric oxide (NO), prostaglandin E2, and IL8, as well as the expressions of inducible nitric oxide synthase (iNOS) and cyclooxygenase (COX)2 without IkB-α degradation and NF-κB activation in cytokine-stimulated human intestinal HT-29 cells [14]. Cyanidin-3-O-sophoroside and cyanidin-3-O-sambubioside from black peanut ameliorated UV-irradiated oxidative injury through the action of the nuclear factor erythroid 2-related factor 2 (NRF2) by interaction with the NF-κB signaling pathway in human keratinocyte cells and mice skin [15]. 

Red clover showed anti-inflammatory effect [16,17]. Although its isoflavones seem to be responsible for this effect [16,17], anthocyanins of red clover may also support this effect. However, the effects of the anthocyanins of red clover on inflammation are not explored yet. Thus, the purpose of this study was to examine anti-inflammatory and antioxidant effects of red clover’s anthocyanins and further identify and quantify anthocyanins and other dietary compounds of red clover. 

## 2. Materials and Methods

### 2.1. Preparation of Anthocyanin Fractions from Red Clover

Red clover (*Trifolium pratense*) flowers were purchased from Starwest Botanicals Inc (Sacramento, CA, USA). Petals of the red clover were cleaned and dried at room temperature. Dried petals (50 g) were extracted with 1 L of 80% aqueous methanol (v/v) by homogenization and sonication [18]. Red clover extract (RC) yield was 13.2%. To isolate red clover anthocyanins fraction (RCA), RC was loaded into a C-18 SPE cartridge (Waters, Milford, MA, USA) and 10 mL of 0.01 N HCl was added to remove sugar, acids, and water-soluble compounds. The cartridge was dried with N_2_ gas for 10 min and then washed with 40 mL of ethyl acetate to remove non-anthocyanin flavonoids. Anthocyanins were eluted with 6 mL of acidic methanol. Solvents of RCA were evaporated using a rotary evaporator (Buchi RE120 Rotovapor, Flawil, Switzerland). RCA yield was 0.36%. RC and RCA were stored at −20 °C until use.

### 2.2. Cell Culture

Mouse monocyte RAW 264.7 cells (ATCC, Manassas, VA, USA) were cultured in RPMI 1640 media supplemented with 10% fetal bovine serum (FBS) in a humidified culture incubator containing 5% CO_2_ at 37 °C. Cells were seeded into 12-well plates (Corning Inc., Corning, NY, USA) at a density of 0.5 × 10^6^ and incubated for 24 h. Media was then replaced to serum-free media and cells were treated with RC or RCA. After incubation for 12 h, cells were stimulated with 1 or 0.5 μg/mL lipopolysaccharide (LPS) (Thermo Fisher Scientific, Waltham, MA, USA) for different hours depending on experiments in the presence and absence of RC or RCA. For the gene expression, cells were treated with 1 μg/mL LPS for 3 h. On the completion of the incubation, total RNA and protein were extracted. Cytotoxicity of RC, RCA, and LPS was determined as previously described [18].

### 2.3. Measurement of Intracellular ROS 

To determine the cellular antioxidant effects of RC and RCA, cellular ROS levels were measured using a 2’-7’-dichlorofluorescein diacetate (DCFDA), a fluorogenic dye. Cells were seeded in a black-24-well plate (Corning Inc) at a density of 2.5 × 10^5^ cells per well and incubated with serum-free culture media containing RC or RCA for 12 h. The cells were then stimulated with LPS for 1 h in the presence and absence of RC or RCA. After cells were washed with 1M HEPES buffer (Thermo Fisher Scientific), they were incubated with phenol red- and serum-free culture media containing 10 μM DCFDA for 1 h at 37 °C in the dark. DCF fluorescence was measured at an excitation wavelength of 485 nm and an emission wavelength of 535 nm using a Biotek Synergy H1 microplate reader (Biotek, Winooski, VT, USA). After the fluorescence was measured, total protein was extracted, and protein concentrations were used to normalize fluorescent intensity. ROS levels were expressed as an arbitrary unit of fluorescence intensity/μg total cell protein. 

### 2.4. Quantitative Polymerase Chain Reaction (qPCR) Analysis

Total RNA was extracted using a Trizol and then cDNA was synthesized using XLAScript cDNA MasterMix (Exella GmbH, Feucht, Germany) according to manufacturers’ instructions. The expression of genes that are involved in the regulation of inflammation and antioxidant was examined using the Fast Start Essential DNA Green Light Master kit (Roche, Indianapolis, IN, USA) in a LightCycler 96 (Roche) [18]. Primer sequences were designed using Primer3 (http://bioinfo.ut.ee/primer3-0.4.0/) and confirmed using a Primer-Blast (NCBI database). Primer sequences were listed in Table 1. Ribosomal protein L 32 (RPL32) was used as the housekeeping gene.

### 2.5. Enzyme-Linked Immunosorbent Assay (ELISA) for TNFα

RAW 264.7 cells were plated into 12-well plates at a density of 0.5 × 10^6^/well and incubated for 24 h. Cells were then treated with 0, 5, 10, and 20 μg/mL of RAC for 12 h and then treated with the same samples and 1 μg/mL of LPS for 3 h. TNFα concentrations in the culture supernatant were measured using an ELISA kit (eBioscience, San Diego, CA, USA) following the manufacturer’s instruction. 

### 2.6. Western Blot Analysis

Total protein extraction and a Western blot analysis were performed as previously described [18]. To examine the inhibitory effects of RCA on translocation of NF-κB between nuclear and cytoplasm, cells had the same pretreatments with RCA described above and then were treated with RCA and 0.5 μg/mL LPS for 1 h [19]. Nuclear and cytoplasmic fractions were obtained using a nuclear extraction kit (Cayman Chemical, Ann Harbor, MI, USA) according to the manufacturer’s instruction. To find each incubation time that produces the highest amount of iNOS, COX2, and p47^phox^ proteins, cells were treated with 0.5 μg/mL LPS during different hours. Based on this result, cells were treated with RCA and LPS for 12 h and for 3 h for determining effects of RCA on iNOS, COX2, and p47^phox^ proteins, respectively after RCA pretreatment. Primary antibodies were used as follows: rabbit anti-mouse COX2, iNOS, and NADPH oxidase 1 (NOX1 (1:1000, ABclonal, Woburn, MA, USA), anti-phospho-p47^phox^, anti-p47^phox^, and NF-κB p65 (1:1000, Thermo Fisher Scientific), TATA-binding protein (1:3000, Thermo Fisher Scientific), and β-actin (1:3000, Sigma-Aldrich, St. Louis, MO, USA). Horseradish-peroxidase-conjugated secondary antibodies (goat anti-rabbit IgG and goat anti-mouse IgG, 1:5000, Invitrogen, Carlsbad, CA, USA) were used. 

### 2.7. Identification and Quantification of Red Clover Polyphenols Using HPLC/ESI-MS Analysis 

Separation, detection, and identification of polyphenols from RC were performed using an HPLC/ESI-MS (Waters) according to the method of Cho et al. [20]. The identified polyphenols were quantified by an HPLC (Waters) [20]. Polyphenols were detected at 330 nm and quantified as daidzein equivalents. Total polyphenols were calculated as the sum of individual polyphenols. Anthocyanins were quantified as delphinidin, cyanidin, petunidin, peonidin, and malvidin glucoside equivalents at 510 nm. Total anthocyanins were calculated as the sum of individual anthocyanin monoglucosides. 

### 2.8. Statistical Analysis

Data were analyzed using a one-way analysis of variance with Tukey’s post hoc analysis (Prism 7.0, Graphpad Software Inc., San Diego, CA, USA). *P* values less than 0.05 were considered significant. Data were presented as mean ± standard deviation.

## 3. Results

### 3.1. RC and RCA Decreased the Expression of Genes Related to Pro-Inflammatory Markers 

**Figure 1:**
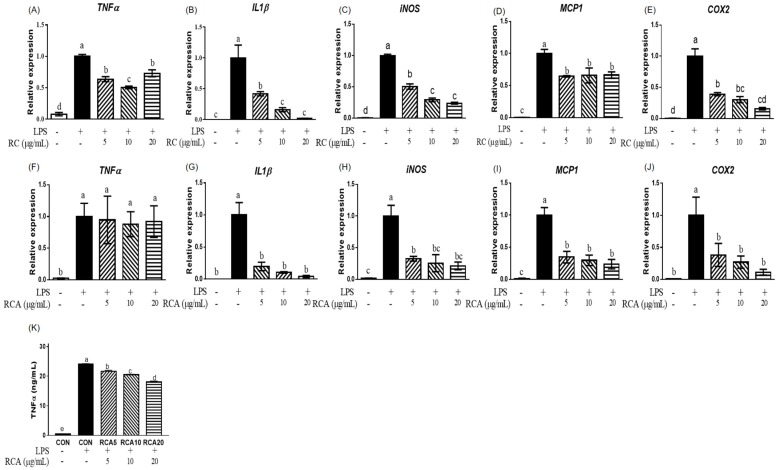
The effect of red clover extract (RC) and red clover anthocyanins fraction (RCA) on gene expression and TNFα secretion in LPS-stimulated RAW 264.7 macrophages. Expression of genes related to pro-inflammatory markers was determined in LPS-stimulated RAW 264.7 cells that were treated with or without RC (**A**–**E**) or RCA (**F**–**J**) at concentrations of 5, 10, or 20 μg/mL. (**K**) Concentrations of TNFα that was released in the media were measured. A different letter indicates a statistically significant difference (*P* < 0.05). + and - indicate the presence and absence of LPS, RC, or RCA, respectively.

To examine the anti-inflammatory effects of RC and RCA, the expression of genes that encode pro-inflammatory markers was measured in LPS-stimulated RAW 264.7 cells with or without treatment of RC or RCA at 5, 10, or 20 μg/mL. LPS increased the expression of *TNFα*, *IL1β*, *iNOS*, monocyte chemoattractant protein (*MCP*)*1*, and *COX2* genes. The LPS-stimulated expressions of *TNFα*, *IL1β*, and *iNOS* genes were attenuated by 5 and 10 μg/mL RC (Figure 1A–C). No additional reduction was observed in the cells that were treated with 20 μg/mL RC. As shown in Figure 1D and E, LPS-induced *MCP1* and *COX2* genes were also downregulated by 5 μg/mL RC up to 64.4% ± 1.1% and 39.9% ± 2.6%, without further reduction in higher concentrations of RC. However, RCA did not alleviate the LPS-induced *TNFα* gene (Figure 1 F). Cells treated with 5 μg/mL RCA exhibited strong suppression on LPS-induced *IL1β*, *iNOS*, and *MCP1* genes (Figure 1G–I). *COX2* gene expression showed a similar suppression at 5 μg/mL RCA compared to the same concentration of RC (Figure 1J). RCA at 10 or 20 μg/mL did not show further inhibitory effect. Although LPS-induced *TNFα* gene expression was not affected by RCA, the secretion of TNFα from macrophage cells into the media was significantly inhibited by RCA in a dose-dependent manner (Figure 1K). The inhibition of 5, 10, and 20 μg/mL RCA was approximately 10%, 15%, and 25%, respectively.

### 3.2. RC and RCA Inhibited LPS-Induced ROS Production

**Figure 2:**
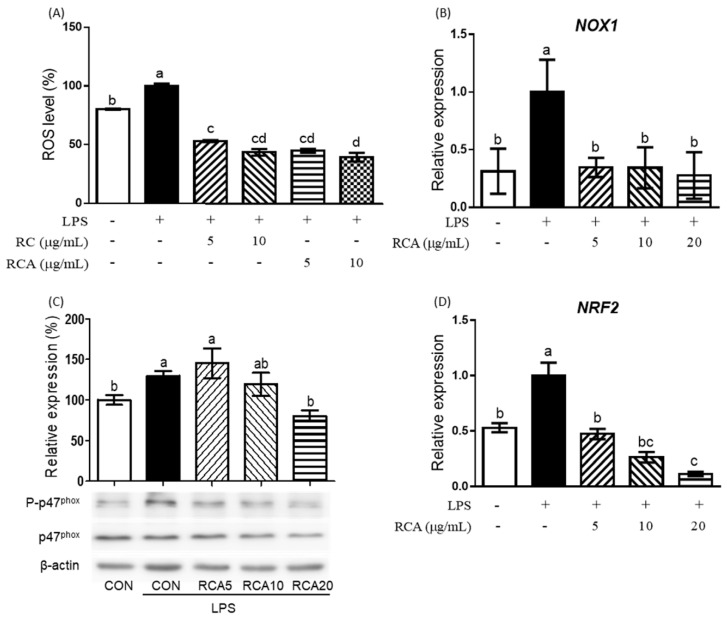
The effect of RCA on intracellular ROS production and the expression of gene and proteins related to oxidative stress. (**A**) Intracellular ROS levels were determined in LPS-stimulated RAW 264.7 cells that were treated with or without 5 or 10 μg/mL RC or RCA. *NOX1* (**B**) and *NRF2* (**D**) genes were measured using qPCR. (**C**) p47^phox^ and its phosphorylation were determined using a Western blot. A different letter indicates a statistically significant difference (*P* < 0.05). + and - indicate the presence and absence of LPS, RC, or RCA, respectively.

LPS produced a significant amount of cellular ROS in RAW 264.7 cells (Figure 2A). RC or RCA at 5 and 10 μg/mL inhibited LPS-induced ROS production to the lower level than that of control cells that were not stimulated by LPS. 

To investigate the mechanism by which RCA inhibits LPS-induced ROS production, the expression of genes and proteins that are involved in the regulation of ROS production were determined (Figure 2B–D). *NOX1* gene encoding a major producer of ROS was significantly upregulated by LPS, whereas this induction was completely prevented in the cells that were treated with 5 μg/mL RCA (Figure 2B). When RAW 264.7 cells were incubated with LPS for 3 h, the strongest phosphorylation on p47^phox^, a 47 kDa cytosolic subunit of NOX1 protein was observed, which was reduced by the treatment of different concentrations of RCA (Figure 2C). RCA at 20 μg/ml showed the most significant inhibition. *NRF2*, a transcriptional factor that regulates response to oxidative stress and inflammation was also induced by LPS (Figure 2D). RCA at 5 μg/mL reversed the expression of LPS-induced *NRF2* gene to the same expression level of the cells that were not stimulated by LPS. RAC at 10 and 20 μg/mL suppressed the expression of *NRF2* gene to the lower level than that of cells that were not stimulated by LPS.

### 3.3. RCA Inhibited the Activation of NF-kB in LPS-Induced RAW 264.7 Cells 

**Figure 3:**
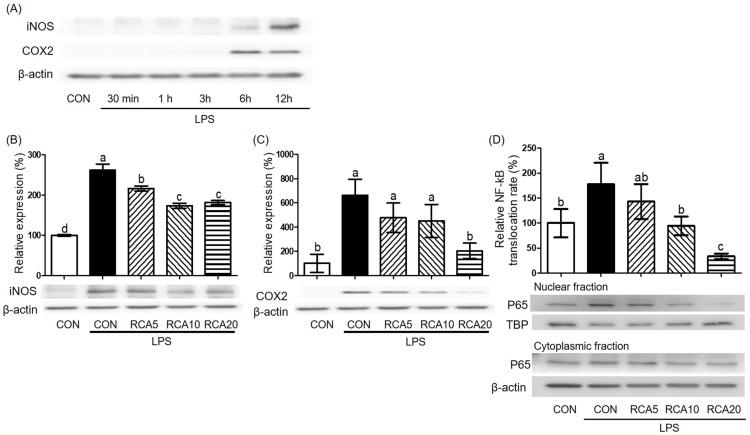
The effect of RCA on NF-kB, iNOS, and COX2 proteins. RAW 264.7 cells were pre-incubated with 5, 10, or 20 μg/mL RCA and then stimulated by 0.5 µg/mL LPS in the presence or absence of RCA. (**A**) RAW 264.7 cells were stimulated by 0.5 µg/mL LPS for 30 min, 1, 3, 6, and 12 h and then iNOS and COX2 proteins were measured. (**B**) iNOS and (**C**) COX2 proteins were determined in RAW 264.7 cells after 12 h-LPS exposure in the presence or absence of RCA. (**D**) p65 of NF-κB protein was determined in nuclear and cytoplasm of RAW 264.7 cells after 1 h-LPS exposure in the presence or absence of RCA. TBP and β-actin proteins were used as housekeeping proteins in nuclear and cytoplasm, respectively. A different letter indicates a statistically significant difference (*p* < 0.05).

To decide LPS-incubation time that produced the highest amount of iNOS and COX2 proteins, RAW 264.7 cells were incubated with LPS for 30 min, 1, 3, 6, and 12 h and then proteins were examined. The highest induction of both iNOS and COX2 proteins was observed after a 12 h-LPS-exposure (Figure 3A). Under this condition, LPS-stimulated iNOS protein was decreased by 5 and 10 µg/mL RCA, but there was no further reduction in cells treated with 20 µg/mL RCA (Figure 3B). LPS-stimulated COX2 protein was almost completely abolished in the cells that were treated with 20 μg/mL RCA (Figure 3C). 

To examine whether the RCA inhibited genes and proteins of pro-inflammatory markers by regulating activation of NF-κB, activation of NF-κB that is indicated by translocation of p65 protein, a subunit component of NF-kB from the cytosol to nucleus was determined by measuring the relevant amount of p65 protein in cytosol and nucleus of cells that were treated with or without LPS in the absence or presence of RCA (Figure 3D). p65 protein was relatively increased in the nuclear when the cells were stimulated by LPS, compared to that in the cytosol. The increased translocation in the nuclear was attenuated by RCA. 

### 3.4. Identification and Quantification of Red Clover Polyphenols

As shown in Table 2, 27 peaks were identified from RC at the wavelength of 330 nm. Individual compounds were reported as µg daidzein equivalents/g of RC. The peak eluting at 37 min which showed the highest concentration (24560.7 ± 60.7 µg daidzein equivalents/g of RC, 449,287 m/z) was unknown tetrahydroxyflavone glucoside, followed by a peak eluding at 38.9 min (17933.8 ± 123.6 µg daidzein equivalents/g of RC, 519,271 m/z) which was identified as gemosteom-7-O-β-D-glucoside-6″-malonate. Other abundant polyphenols having concentrations of more than 10,000 µg daidzein equivalents/g of RC were confirmed as genistin (peak 6,12892.3 ± 35.6 µg daidzein equivalents/g of RC, 433,271,153 m/z) and kaempferol or luteolin (peak 16 12876 ± 16.1 272 m/z) 

Anthocyanins were identified at a wavelength of 550 nm (Table 3). Individual compounds were reported as µg cyanidin-3-glucoside, delphinidin-3-glucoside, petunidin-3-glucoside, peonidin-3-glucoside, and malvidin-3-glucoside equivalents per g of RC. Based on the quantification of an individual compound, malvidin-3-O-galactoside was a major anthocyanin of RC, being eluted at 36.3 min (peak 6, 2129.4 ± 6.6 malvidin-3-glucoside equivalents/g of RC, 493,331 m/z). As the second abundant molecule of anthocyanin in RC, peonidin-3-O-monogalactoside was identified in a peak 5 eluting at 34.4 min (633.6 ± 5.5 peonidin-3-glucoside equivalents/g of RC, 463,301 m/z). 

## 4. Discussion

This study determined anti-inflammatory and antioxidant effects of anthocyanins of red clover using LPS-stimulated macrophage cells and identified and quantified anthocyanins and other dietary compounds of red clover. RC that was extracted using DMSO inhibited the secretion of TNFα and IL6 and suppressed iNOS, COX2, and NF-κB proteins in LPS-stimulated RAW 264.7 cells [17]. Muller et al. showed that isoflavones such as biochanin A, genistein, and daidzein were responsible for the anti-inflammatory effect of red clover [17]. In this study, both RC and RCA suppressed LPS-induced *IL1β*, *iNOS*, *MCP1*, and *COX2* genes. This indicates that anthocyanins of red clover may be critical for the anti-inflammatory effects of red clover. RC reduced LPS-induced *TNFα* gene expression in this study, but this gene was not changed by RCA. Interestingly, without a change in *TNFα* gene expression, the secretion of TNFα was suppressed by RCA. Chemokines, e.g., TNFα are regulated by post-transcriptional regulation by controlling RNA stability and/or translational regulation [21]. Mice deleting TNF AU-rich elements (ARE) in the 3´-untranslated region of a transcript encoding TNFα exhibited increased secretion of TNFα by decreasing a rate of TNFα decay and translational repression in hemopoietic and stromal cells [22]. Therefore, RCA may suppress LPS-induced TNFα secretion by controlling translational inhibition without a change in RNA stability. Consistent with RCA-suppressed genes and proteins related to cytokines and inflammatory enzymes, translocation of p65 subunit of NF-κB into the nucleus was inhibited by RCA in this study. This indicates that the anti-inflammatory effects of anthocyanins of red clover may be mediated by regulating the NF-κB signaling pathway.

In response to LPS-induced cytokines, iNOS and COX2 catalyze the production of NO and prostaglandin E2, respectively, which are potent pro-inflammatory mediators [23]. In this study, LPS-induced both iNOS and COX2 genes and proteins were suppressed by RCA. The iNOS positively regulates NOX which produces superoxide by transferring electrons from NADPH to oxygen [24,25]. Under stimulated conditions, e.g., LPS, NOX enzymes are activated by protein–protein interactions via its cytosolic subunits (p47^phox^, p67^phox^, and p40^phox^) and membrane subunits (small-G-protein, Rac1, or Rac 2) and by phosphorylation of p47^phox^ [26]. Moreover, p47^phox^ is a critical organizer to bring these subunits to complexes by its localization to the membrane and its phosphorylation-induced conformational changes on the protein [25]. In this study, LPS-induced NOX1 gene and phosphorylation on p47^phox^ were completely abolished by RCA treatment at 5 and 20 µg/mL, respectively, which supports reduced intracellular ROS production. Because LPS-induced NOX1 is NF-κB dependent [27], our findings indicate that RCA modulates cellular oxidative stress by suppressing NOX1 through reduced NF-κB.

When ROS increases in the body, the antioxidant system is promoted to remove free radicals. NRF2 is a major transcriptional factor to regulate genes and proteins that are involved in the antioxidant system. LPS-stimulated RAW 264.7 cells that were treated with berry anthocyanins showed a significant decrease in NRF2 and its downstream genes including catalase and superoxide dismutase [28]. Extracts from red clover showed high antioxidant activities using ABTS radical, DPPH radical, hydrogen peroxide, and superoxide radical scavenging assays [29], which may be effective at cellular levels. In this study, LPS-induced ROS production led to an increase in *NRF2* gene expression, which activates cellular antioxidant systems to remove ROS. *NRF2* gene expression was reduced by RCA, due to possibly either less ROS production by decreased NOX1 or anthocyanins’ antioxidant activities. Consistent with this, NOX1 gene and phosphorylation of p47^phox^ were reduced to the basal level of cells untreated with LPS and samples, but LPS-stimulated cells with RC or RCA exhibited lower ROS levels than the basal level, suggesting that further ROS reduction beyond the NOX1 and its regulation observed in this study may be caused by the antioxidant activity of red clover, especially its anthocyanins [29]. Therefore, its anthocyanins may save NRF2 activity by directly removing ROS using its strong antioxidant capacity.

Some dietary compounds that are previously found in red clover [30], were also identified in this study, but additional dietary compounds were identified, which may be due to detection at a different wavelength. Cyanidin-3-O-sophoroside and cyanidin-3-O-sambubioside were identified as major anthocyanins from red clover using thin-layer chromatography [31]. However, these compounds were not identified in this study using LC-MS. This discrepancy results from different methodologies and approaches for extraction and identification. In this study, malvidin-3-O-galactoside was the most abundant anthocyanin of red clover. Although malvidin-3-glucose had a higher anti-inflammatory effect than malvidin-3-O-galactoside, malvidin-3-O-galactoside also suppressed the expression of MCP1, intracellular adhesion molecule-1, and vascular cell adhesion molecule-1 genes and proteins by inhibiting IkB degradation and translocation of p65 protein of NF-kB in TNFα-stimulated human umbilical vein endothelial cells [12]. Other anthocyanins detected in the red clover including peonidin-3-O-monogalactoside, cyanidin-3-O-monogalactoside, cyanidin-3-O-monoglucoside, petunidin-3-O-monogalactoside, delphinidin-3,5-O-diglucoside, and petunidin-3-O-rutinoside are also found in blueberry, concord grape, and myrtle berry, which have strong antioxidant and anti-inflammatory effects [28,32,33]. 

## 5. Conclusions

In summary, anthocyanins of red clover inhibit LPS-induced inflammation and oxidation in macrophages in vitro. This study only tested RC and RCA’s effects on RC and RCA-pretreated cells, followed by LPS stimulation. Therefore, this indicates RC and RCA’s protective effects on acute LPS-stimulated inflammation and oxidation. Further studies taking into account pre-existing inflammation in vitro and in vivo using animals and humans such as arthritis, Crohn’s disease, are needed to clarify anti-inflammatory and antioxidant effects of red clover and its anthocyanins. 

## Figures and Tables

**Table 1:** Primer sequences for qPCR.

Gene	Forward	Reverse
*COX2*	GCCTACTACAAGTGTTTCTTTTTGCA	CATTTTGTTTGATTGTTCACACCAT
*GAPDH*	GGTGGTCTCCTCTGACTTCAACA	GTTGCTGTAGCCAAATTCGTTGT
*IL1β*	GTCACAAGAAACCATGGCACAT	GCCCATCAGAGGCAAGGA
*iNOS*	AATCTTGGAGCGAGTTGTGG	CAGGAAGTAGGTGAGGGCTTG
*MCP1*	CTTCTGGGCCTGCTGTTCA	CCAGCCTACTCATTGGGATCA
*NOX1*	TTCACAGTTATTCATATCATTGC	AGAGAACAGAAGCGAGAG
*NRF2*	CTCGCTGGAAAAAGAAGTG	CCGTCCAGGAGTTCAGAGG
*TNFα*	GGCTGCCCCGACTACGT	ACTTTCTCCTGGTATGAGATAGCAAAT

**Table 2:** Identification and quantification of isoflavones of red clover.

Number	RT (min)	Isoflavone Derivatives	Amount (μg/g dw)	[M-H]-m/z
1	27.1	Luteolin 7-O-β-D-glucoside	368.5 ± 14.7	449, 287
2	28.7	unknown tetrahydroxyflavone glucoside	2394.3 ± 28.0	449, 287
3	29.2	unknown tetrahydroxyflavone glucoside	1356.2 ± 18.0	449, 287
4	31.5	Isoquercitrin-6″-O-malonate	1677.9 ± 20.9	551, 303
5	32.3	Pratensein-7-O-β-D-glucoside	682.1 ± 6.3	463, 301
6	33.4	Genistin	12892.3 ± 35.6	433, 271, 153
7	33.9	Hyperoside	3103.0 ± 20.9	465, 303
8	34.3	Isoquercetriin	8940.1 ± 16.6	465, 303
9	35.0	Apigenin-7-O-β-D-glucoside	512.1 ± 1.7	433, 271
10	36.1	Pseudobaptigenin	5809.6 ± 14.0	283
11	37.0	unknown tetrahydroxyflavone glucoside	24560.7 ± 60.7	449, 287
12	38.4	Kaempferol or Luteolin	5022.7 ± 37.8	287
13	38.9	Gemosteom-7-O-β-D-glucoside-6″-O-malonate	17933.8 ± 123.6	519, 271
14	39.3	unknown tetrahydroxyflavone glucoside	2691.2 ± 13.6	449, 287
15	40.2	3-methylquercetin-7-O-β-D-glucoside	7838.7 ± 7.6	479, 317
16	42.7	Kaempferol or Luteolin	12876.9 ± 16.1	287
17	44.3	Pratensein-7-O-β-D-glucoside-6″-malonate	2437.5 ± 8.8	549, 317
18	44.9	Pseudobaptigenin-7-O-β-D-glucoside	2280.1 ± 8.4	445, 283
19	45.5	Kaempferol or Luteolin	2213.7 ± 9.1	287
20	46.7	Glycitein	312.1 ± 15.1	285, 167
21	47.5	Pseudobaptigenin-7-O-β-D-glucoside-6″-O-malonate	2361.1 ± 47.3	445, 283, 137
22	52.2	Formononetin-7-O-β-D-glucoside-6″-O-malonate	2231.1 ± 19.0	517, 269
23	53.7	Calysosin-Glucoside-Malonate	213.7 ± 15.9	533, 285, 137
24	57.0	Prunetin-4′-O-β-D-glucoside-6″-O-malonate	2089.3 ± 38.1	533, 285
25	58.7	Formononetin	497.3 ± 13.6	269, 137
26	60.2	Biochanin A-7-O-β-D-glucoside-6″-O-malonate	3713.8 ± 3.1	533, 285
27	66.8	Biochanin A	1059.8 ± 14.8	285
Total			128069.5 ± 628.9	

Values represent means ± standard deviation, RT: retention time, dw: dried weight.

**Table 3:** Identification and quantification of anthocyanins of red clover.

Number	RT (min)	Anthocyanins	Amount (μg/g dw)	[M-H]-m/z
1	27.5	Delphinidin-3, 5-O-diglucoside	139.0 ± 1.1	627, 465, 303
2	29.9	Cyanidin-3-O-galactoside	380.3 ± 2.2	449,287
3	31.5	Cyanidin-3-O-glucoside	47.7 ± 3.3	449,287
4	33.5	Petunidin-3-O-galactoside	145.5 ± 4.4	479,317
5	34.4	Peonidin-3-O-galactoside	633.6 ± 5.5	463,301
6	36.3	Malvidin-3-O-galactoside	2129.4 ± 6.6	493,331
7	37.3	Petunidin-3-O-rutinoside	123.9 ± 7.7	625,479,317
Total			3599.5 ± 10.4	

Values represent means ± standard deviation, RT: retention time, dw: dried weight.
